# Editorial: Stroke and infarction at high-altitude

**DOI:** 10.3389/fphys.2022.1114747

**Published:** 2022-12-09

**Authors:** Esteban Ortiz-Prado, Francisco C. Villafuerte, Julien Vincent Brugniaux, Juan Izquierdo-Condoy, Ginés Viscor

**Affiliations:** ^1^ One Health Research Group, Faculty of Medicine, Universidad de Las Américas, Quito, Ecuador; ^2^ Laboratorio de Fisiología Comparada-LID/Fisiología del Transporte de Oxígeno-IIA, Universidad Peruana Cayetano Heredia, Lima, Peru; ^3^ INSERM 1300, Université Grenoble-Alpes, Grenoble, France; ^4^ Dirección Nacional de Inteligencia de la Salud, Ministerio de Salud Púbica, Quito, Ecuador; ^5^ Secció de Fisiologia Departament de Biologia Cel·lular, Fisiologia i Inmunologia, Universitat de Barcelona, Barcelona, Spain

**Keywords:** hypoxia, stroke, infarction, high altitude, editorial

Cardiovascular and cerebrovascular disease, including ischemic heart disease (IHD) and cerebrovascular events such as hemorrhagic or ischemic stroke, are the leading causes of premature death and disability worldwide (Feigin et al., 2021). At least 5.7% of the world’s population lives at relatively high altitude locations (above 1,500 m); however, the association between life at high altitude exposure and IHD or stroke has been poorly studied. The progression towards cardiovascular and cerebrovascular disease is particularly relevant to high-altitude populations given the hypoxic environmental conditions and their vascular and hematological characteristics. Although epidemiological studies have shown relevant cardiovascular and neurological findings in these populations, it is unclear how high-altitude hypoxia and the characteristic elevated hematocrit are associated with cardio-cerebrovascular disease. It is still uncertain and intricate, for example, to conclude about the role of direct or indirect effects of systemic hypoxia on stroke or IHD risk factors such as endothelial dysfunction and damage, thrombogenesis, hypercoagulability, atherosclerosis, platelet aggregation, or onmodifiable factors related to these diseases (Tymko et al., 2019; Ortiz-Prado et al., 2022). It is well known that in high-altitude populations, chronic exposure to hypoxia over several generations leads to the selection of a number of adaptive characteristics to this condition, whereas lowlanders who are exposed acutely or live for long periods at high altitude, even during their lifespan, become acclimatized (Ortiz-Prado et al., 2010; Moore et al., 2011). Elucidating whether adapted or acclimatized humans are at lower or higher risk of developing cardiovascular disease is complex, in part due to the vast number of molecular, genetic, physiological, and clinical factors that vary from population to population, but also to the fact that most people tend to live and visit high-altitude locations, work shifts and migrate from time to time, therefore, assessing and controlling for the severity of their actual hypoxic exposure regime is difficult (West, 2012). In this sense, Frontiers in Physiology has made an effort to put out an editorial call for papers seeking to explore the role of hypoxia on the development of different factors associated with a lower or higher risk of developing ischemic or hemorrhagic- related events, such as those affecting the brain or the heart.

The first manuscript by Ortiz-Prado et al. tries to elucidate the differences in terms of mortality and incidence of cerebrovascular events in different populations located in Ecuador (Ortiz-Prado et al.). This study, which included more than 75,000 patients and more than 38,000 deaths in a range of 0–4,300 m of altitude, may serve as an indirect indicator of the protective effect that living at high altitudes may have on cerebrovascular events. The authors also speculated and discussed the protective role and its variability as one ascends to higher altitudes where prothrombotic factors may outweigh protective factors such as angiogenesis. The authors concluded that living at high altitudes could be protective regarding mortality and disability. Still, this effect only remains evident between 1,500 and 3,500 m of altitude, and above these elevations, the risk rises again (Ortiz-Prado et al.). In this same line, another interesting work from Gonzalez-Candia and Herrera sought to review and analyze the possible long-lasting postnatal effects that chronic hyperbaric hypoxia might have on maternal, placental, and fetal adaptive responses. They concluded that studies on Andean populations have shown a number of negative effects of high altitude, although these populations have been able to create adaptations to the thin air. Pathologies such as systemic hypertension, hemorrhage, oligohydramnios, placental insufficiency, and premature delivery have been proposed as triggers of pregnancy complicationsat altitude. Gonzalez-Candia and Herrera have compiled information on the possible role of high-altitude exposure on maternal and fetal vasodilation capacity. For example, in Andean populations, long-term exposure to hypoxia suggests more efficient O_2_ transfer and use, determined by decreased lung volume, less pulmonary vasoconstriction, higher uterine artery blood flow, and better cardiac O_2_ utilization relative to lowlanders. Despite the information available, the physiological processes contributing to fetal growth restriction and subsequent health or disease programming at altitude still need to be better understood. Thus our ability to intervene remains limited. This Research Topic also received two additional investigations focusing on determining the difference in certain biochemical markers at different altitudes. Chronic mountain sickness (CMS, Monge’s disease), a specific condition affecting 5–33% of high-altitude dwellers, is characterized by severe hypoxemia, excessive erythrocytosis (EE), and a range of associated clinical symptoms (Champigneulle et al., 2022). In this sense, Schmidt et al. presented a study that analyzed hemoglobin mass, plasma, and blood volume in healthy residents living at high altitudes and compared them to those suffering from CMS and individuals with no previous pathologies. The study included highlanders (∼3,900 m), of which 34 had CMS, 11 had excessive erythrocytosis without other signs of CMS (Hb concentration greater than 21 g/dl), 20 were healthy highlanders, and 22 lowland residents (420 m) as the control group, and analyzed hemoglobin mass (Hbmass), plasma volume (PV) and blood volume (BV) data, quantifying the dependence of hemoglobin concentration on Hbmass and PV (Schmidt et al.). The authors concluded that the substantial increase in hemoglobin concentration was dependent on two factors: an increase in Hb mass (accounting for ∼65% of theincrease) and a reduction in plasma volume (accounting for ∼35%). The last study by Turton et al., analyzed pro-coagulation factors and their role in hypercoagulation in healthy volunteers after rapid ascents to high altitudes. The study’s objective was to investigate the relationship between soluble P-selectin (a marker of platelet activation) and von Willebrand factor (vWF) upon exposure to hypoxia. Plasma concentrations of P-selectin and vWF were measured in healthy volunteers before, during, and after the APEX 2 expedition (Turton et al.). Sixteen participants residing at 600 m (who had not been to altitudes above 1,500 m in the previous 3 months) were recruited, and baseline samples were taken at this location. They ascended by plane to 3,600 m (where they spent 5 days) and then ascended by road for 90 min to 5,200 m, where blood samples were taken on days 1, 3, and 7 of a 7-day stay. Positive control (recovery) samples were taken 11 weeks after the expedition. Healthy, young adult participants showed that high-altitude significantly increased mean plasma P- selectin and vWF compared to pre-expedition levels; nevertheless, both plasma markers returned to baseline after the expedition. These results are consistent with previous work showing evidence of platelet activation at high altitudes and demonstrated that the increase in P-selectin is not simply due to an increase in platelet count (Shang et al., 2019; Wang et al., 2020; Wang et al., 2022). Certain prothrombotic and pro-coagulation markers rise after rapid exposure to high altitude, and this may indirectly be associated with the increased development of various ischemic problems, whether cardiac or neurological. This editorial goes along the idea to provide the reader with a broader perspective on the role that altitude may play in different pathological neurological or cardiovascular conditions, as well as the molecular and physiological responses that might be linked to higher hematocrit (higher [Hb]) that might be associated with stroke or cardiovascular risks.
